# Photobiomodulation therapy reduces postoperative pain after third molar extractions: A randomized clinical trial

**DOI:** 10.4317/medoral.24228

**Published:** 2020-12-19

**Authors:** Cristina Pereira Isolan, Mateus de Azevedo Kinalski, Otávio Amaral de Andrade Leão, Letícia Kirst Post, Tania Maria Pereira Isolan, Mateus Bertolini Fernandes dos Santos

**Affiliations:** 1 Post-doc, Graduate Program in Dentistry, Federal University of Pelotas, RS, Brazil; 2 PhD Candidate, Post-Graduate Program in Dentistry, Federal University of Pelotas, RS, Brazil; 3 PhD Candidate, Post-Graduate Program in Epidemiology, Federal University of Pelotas, RS, Brazil; 4 Associate Professor, Graduate Program in Dentistry, Federal University of Pelotas, RS, Brazil; 5 Private Practice, Pelotas, RS, Brazil

## Abstract

**Background:**

To assess the efficacy of PBMT on reducing postoperative pain scores in patients submitted to third molar extractions.

**Material and Methods:**

A randomized controlled trial (ReBEC:RBR-94BCKZ) was designed according to the SPIRIT and followed the CONSORT. Patients were randomly allocated according to control or PBMT groups. PBMT consisted of the application of GaAlAs laser (808nm;50mW) applied in six points (1.23 min;11 J/cm2) after extraction. Pain scores were assessed using the Visual Analogue Scale (VAS) in millimeters evaluated after 6 (T6), 24 (T24), and 48 (T48) hours. The Wilcoxon Mann–Whitney test was used to check for possible associations between VAS scores and treatment groups.

**Results:**

A total of 101 third molar extractions were performed in 44 patients. The mean age was 28 years old(SD±11.54). Comparing control and intervention, PBMT group showed a significant effect on the reduction of postoperative pain at T6(mean VAS=0.9; C.I:0.63–1.16) compared to control (mean VAS=2.5;C.I:2.1–2.88)(*p*<0.001). The same statistically significant effect on the reduction of postoperative pain was observed at T24 (PBMT mean VAS=0.72;C.I:0.51–0.93; control mean VAS=2.86;C.I:2.40–3.31;*p*<0.001) and T48 (PBMT mean VAS=0.64;C.I:0.36–0.92; control mean VAS=2.86;C.I:2.37–3.34;*p*<0.001).

**Conclusions:**

PBMT significantly reduce the postoperative pain scores when assessed 6, 24, and 48 hours after third molar extractions.

** Key words:**Controlled clinical trial, gallium aluminium arsenide lasers, third molar.

## Introduction

Third molars extraction is a common dental procedure both in private practice and hospital settings. This type of procedure often requires a considerable involvement of the surrounding connective tissues and, depending on tooth position, it might be necessary to conduct bone removal or tooth sectioning, which could increase invasiveness, surgical time, and are often related to increased morbidity, that could result in impairment of daily activities during the postoperative period, due to reported discomfort, swelling, trismus, and postoperative pain (1,2).

The standard therapeutic approach to pain management is the administration of analgesic drugs, corticosteroids, or nonsteroidal anti-inflammatory drugs (NSAIDs) (3). Previous studies reported that the use of NSAIDs prior to the procedure could slightly reduce pain scores during the postoperative period and that corticosteroid administration also could be effective in reducing pain after third molar extractions (4,5). However, the absence of a standardized protocol could result in indiscriminate use of such medications, that might affect patient’s general health, since those drugs usually present side effects in renal, gastrointestinal, and cardiovascular systems (6,7).

In this perspective, several alternatives have been reported aiming to reduce postoperative pain, such as cryotherapy, use of Platelet-Rich Fibrin (PRF), and photobiomodulation therapy (PBMT) (8-10). The PBMT mechanism cconsists in the application of infrared light that is absorbed by the cells and tissues promoting a chemical change that aims to reduce inflammatory levels and consequently relieving pain (11). Although the PBMT might be an appropriate approach for reducing pain after surgery, a lack of evidence still exists considering third molars removal (12).

The decision-making process in healthcare procedures must consider high-quality evidence studies to guide the interventions, and randomized clinical trials are one of the greatest levels of evidence considering the hierarchical pyramid of evidence (13). Thus, the present study aimed to assess the efficacy of PBMT (low-level gallium aluminum arsenide laser therapy with 808nm wavelength) on postoperative pain scores in patients submitted to third molar extractions.

Material and methods

- Study Design

This prospective equivalence randomized controlled trial with parallel-groups blinded to the evaluators was designed following the SPIRIT statement and is reported following CONSORT guidelines. The protocol was approved by the institutional ethics committee (Protocol 3.699.947) and the trial was registered prior its beginning (ReBEC TRIAL: RBR-94BCKZ). The study was conducted from June 2017 to January 2020.

- Eligibility criteria

The inclusion criteria were: 1) Young adults; 2) Indication for third molar removal; 3) Good general health, which allows for surgical removal; 4) Signed informed consent form for participation and permission to use obtained data or research purposes.

Exclusion criteria: 1) Systemic disease that prevents surgery; 2) Continuous use of any medication. Also, patients that reported the use of analgesic after surgery, both in control or experimental group, were excluded of the study.

- Randomization and allocation concealment

Patients were randomly allocated according to the type of intervention (control group: third molar extraction; intervention group: third molar extraction + PBMT) that were performed considering the tooth as unit; the randomization process was performed using the Random Allocator software© in blocks of 6. To ensure concealment of randomization, consecutively numbered brown envelopes were used, with the following intervention draws: CONTROL and PBMT and only one researcher was involved in this process. The surgeon only became aware of the type of intervention at the time of surgery when the envelope was opened by a blinded researcher.

- Clinical procedures

All surgeries were carried out by the same surgeon (T.M.P.I) which is specialist in oral maxillofacial surgery. All patients were orally premedicated with 2g of amoxicillin 1 hour prior to the surgery and were prescribed to take 500mg of amoxicillin every 8 hours for 7 days (14). Local anesthesia was induced by articaine 4% with epinephrine (1:100,000), and the procedures of tooth extraction were conducted following conventional techniques (15). When impacted, access to the teeth was obtained through mucoperiosteal incision, flap elevation, and osteotomies, when needed. The decision of tooth sectioning was made by the surgeon considering specificities of each clinical case. The soft tissue was then carefully repositioned and sutured with a nonabsorbable monofilament suture (No. 5- 0 nylon; Ethicon [Johnson & Johnson], São José dos Campos, Brazil). All patients received standardized information regarding postoperative care and a single dose of analgesic medication (Dipyrone monohydrate 500mg) right after the surgery and were asked to report the use of analgesic intake in the follow-up appointment.

- Study groups

1) Control

The tooth extraction was performed according to the specificities of each clinical case. After tooth removal, the surgical area was cleansed with 0.9% saline solution and sutures were performed.

2) PBMT group

PBMT [Gallium Aluminum Arsenide Diode (GaAlAs)] (wavelength: 808nm, average power density: 50mW, circular spot diameter: 0.71cm, spot area: 0.4cm2) in continuous mode was applied in six points in contact with the soft tissue (1.23 min in each point of application; dose per point 11 J) after the sutures. The application points were divided into two points in the labial region (apical and cervical); two points in the lingual region (apical and cervical); and two points in the previous occlusal direction, resulting in a total dose of 66 J. This PBMT protocol was applied only in the tooth extraction and is based on a previous study (16,17).

- Sample size calculation

The sample size was calculated using an earlier study that assessed different interventions to relief postoperative pain after third molar extractions using visual analog scale (VAS) (18). The sample was calculated using t-test considering an expected difference of 0.08 in VAS means, 0.2 as expected standard deviation, 80% power, and 95% significance level, resulting in a sample size of 100 teeth.

- Primary outcome – Postoperative pain

The primary outcome was the evaluation of pain level using a Visual Analogue Pain Scale (VAS), ranging from zero (no pain) to 10 (worst possible pain) (19). The following postoperative periods were assessed: 6 (T6), 24 (T24), and 48 (T48) hours after surgery.

- Statistical Analyses

Descriptive analyses with mean values and standard deviation (SD) or frequency distribution (%) were calculated for each variable. Statistical analysis was performed using Stata Software 16.0 (Stata Corporation, College Station, TX, USA). Comparison between control and PBMT group between characteristics of tooth extraction were performed through the chi-square test. Data were tested for normality by means of a Shapiro-Wilk test and found to not be normally distributed. The Wilcoxon Mann–Whitney test was used to check for possible associations between VAS scores and treatment groups. Statistical significance was set at the alpha level of 0.05.

## Results

**Figure 1:**
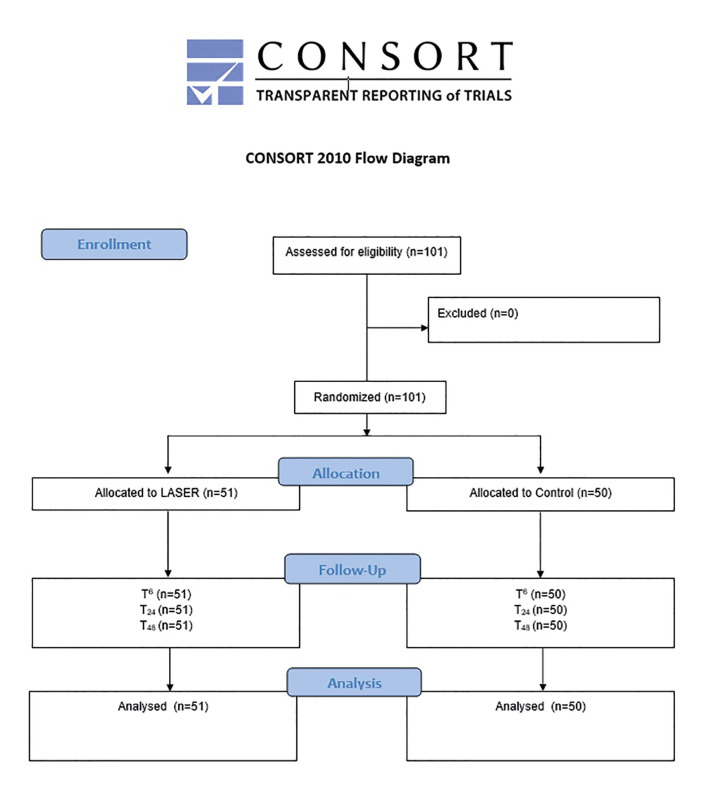
CONSORT flow diagram.

The CONSORT flow diagram with the enrollment characteristics of this study is presented in Fig. 1.

A total of 101 third molar extractions were performed in 44 patients according to the randomization process. The sample was composed in its majority by females (n=28 / 63.6%), and most of the patients did not have hypertension (93.2%), smoking habits (100%), and diabetes (100%). The mean age of the sample was 28 years old (SD ± 11.54). The characteristics of the sample regarding the patients and extracted teeth are presented in Table 1, respectively. It is also possible to observe that there were no differences in the distribution of teeth region, Pell and Gregory position and classification, included or impacted and the need of osteotomy or tooth sectioning between the control and PBMT groups.

When considering the whole sample, the average VAS pain scores were 1.69 at the first assessment (T6), increased a little 24 hours after the intervention [1.78] and then reduced slightly in the last assessment (1.74 at T48). Table 2 compares the postoperative VAS pain scores between PBMT and control groups considering different postoperative periods. The PBMT showed a statistically significant effect (*p*<0.001) on the reduction of postoperative pain at T6 where PBMT VAS mean score was 0.9 (0.63–1.16) compared to a mean VAS score of 2.5 (2.11–2.88) in the control group. The same statistically significant effect on the reduction of postoperative pain was observed at T24 (0.72 for PBMT and 2.86 for control group; *p*<0.001) and T48 (0.64 for PBMT and 2.86 for control group; *p*<0.001).

Table 3 and Table 4 compare the Pell and Gregory Position and Classification postoperative VAS pain scores between PBMT and control groups, respectively. Considering the position, the PBMT showed statistically significant differences among the complete postoperative periods (T6, T24, and T48) and at each position (Position A, Position B, and Position C) (*p*<0.001) compared to the control group. According to the classification, the PBMT demonstrated a statistical significance compared to the control group at Class I (*p*=0.02) and Class II (*p*<0.001) at T6, T24, and T48.

**Table 1:**
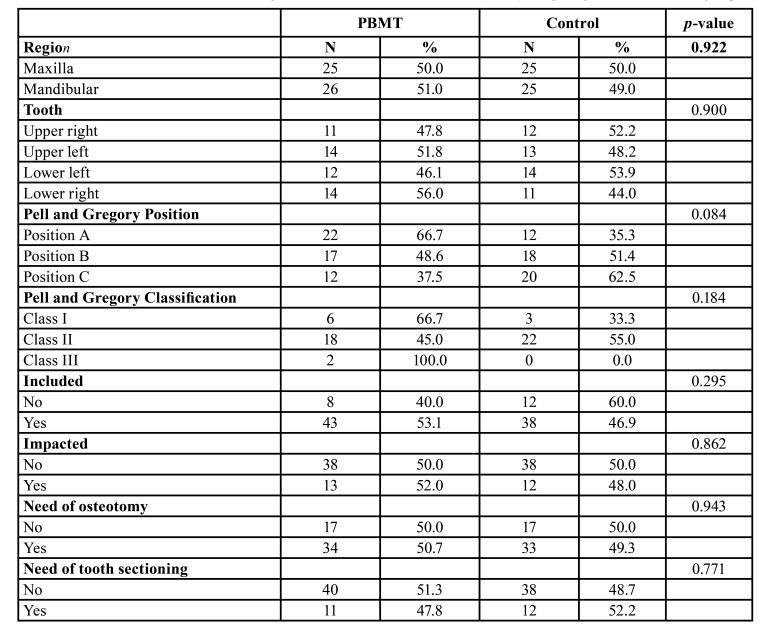
Characteristics of tooth considering the total of third molar extractions (n=101) comparing PBMT and control group.

**Table 2:**
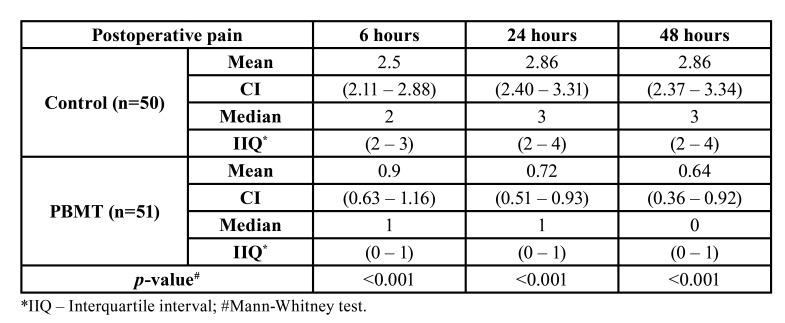
Postoperative pain scores (6h, 24h, and 48h) according to the Visual Analogue Scale (VAS) compared between PBMT and control groups.

**Table 3:**
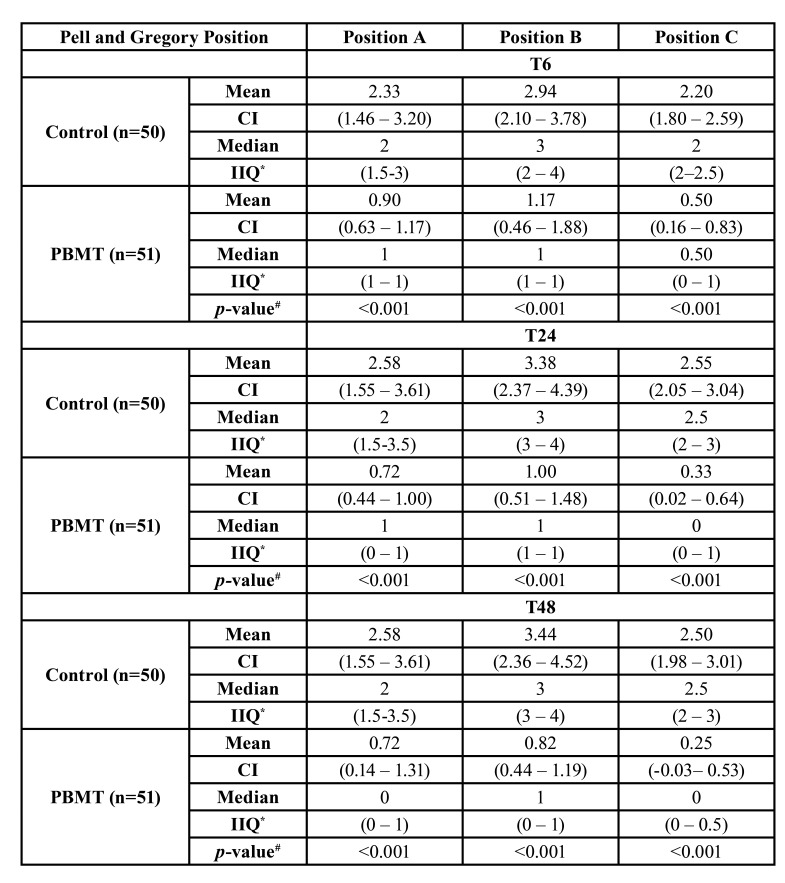
Comparison between the Pell and Gregory Position and Postoperative pain scores (6h, 24h, and 48h) according to the Visual Analogue Scale (VAS) between PBMT and control groups.

**Table 4:**
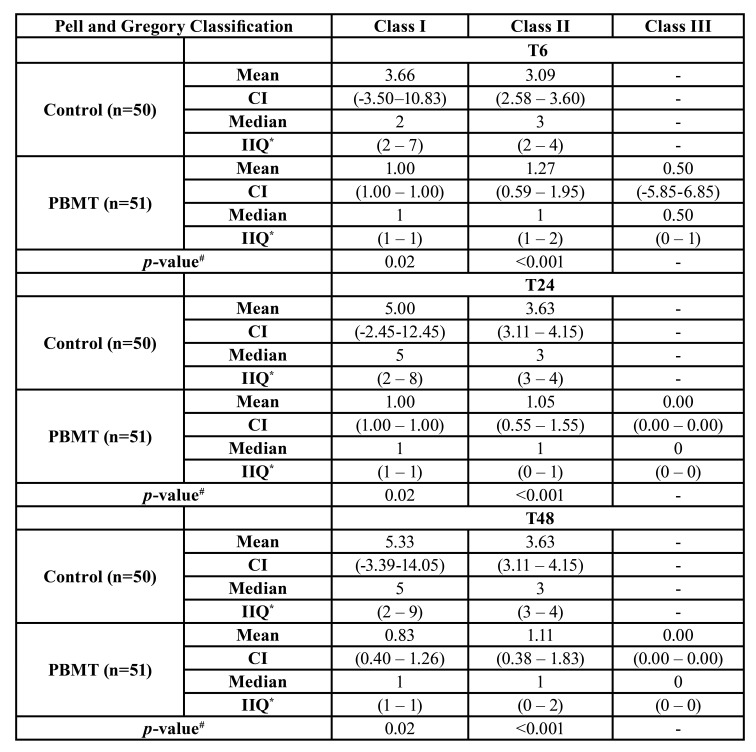
Comparison between the Pell and Gregory Classification and Postoperative pain scores (6h, 24h, and 48h) according to the Visual Analogue Scale (VAS) between PBMT and control groups.

## Discussion

This clinical trial evaluated the PBMT (low-level gallium aluminum arsenide laser therapy with 808nm wavelength) effectiveness on reducing postoperative pain scores in patients submitted to third molar extractions. As previously mentioned, third molar extractions are among the most frequent oral surgery procedures, which is also commonly associated with postoperative pain, resulting in impaired quality of life during the postoperative course. In the literature, there is still a lack of evidence regarding PBMT effectiveness in reducing third molar extraction postoperative pain, where previous studies presented positive and others no difference compared to a control group (20-22). Also, there are no standardized protocols of PBMT considering the number of sessions, administration mode, and wavelengths (23). The findings of our randomized controlled trial add to the literature since it demonstrated a statistically significant reduction of postoperative pain scores when PBMT was performed compared to a control group. The positive effect of PBMT in reducing pain was observed in all three postoperative assessments made in the study (6, 24 and 48 hours after the procedure). In agreement with a previously conducted study, our randomized controlled trial performed only a single session of PBMT also presented the benefits were evidenced during the postoperative period (22). Besides PBMT showed efficacy to reduce pain values comparing different Pell and Gregory positions and classifications of third molar removal. Although no patient was excluded from our study due to reported use of analgesic medication, we have to interpret such findings with caution since patients might not report the use of analgesic when asked, which should be considered a limitation of the present study.

In the last decade, different PBMT specs and protocols have been studied for different outcomes, among them the reduction of pain related to orthodontic treatments, temporomandibular disorders, and recently dental implant stability and peri-implant alterations (24).

The Gallium and Aluminum Arsenide Diode (GaAlAs) laser that was used in the present study has a continuous emission and a wavelength of 620 to 830 nanometers (nm), which is suggested to improve bone healing and analgesic induction through endogenous opioid release (25). Right after a third molar extraction, a repair process initiates by the release of several growth factors, cytokines, and inflammatory mediators, where the PBMT could act to reduce these impacts (26).

A previous systematic review of clinical trials revealed that the PBMT could have positive effects on pain after tooth extraction (27). In our analysis, PBMT showed a statistically significant effect immediately after surgery compared to the control group (*p*<0.001). The same statistically significant effect could be observed at 24 and 48 hours on the reduction of postoperative pain.

Pain relief is an important outcome associated with the quality of life of subjects submitted to oral surgeries and these consequences could impair daily activities of these subjects including psychological, physical, and social aspects (28). Although the Quality of Life outcome was not directly assessed in this study, another recent review study suggested that the main negative impact is usually observed in the first postoperative day, decreasing in the following days (29). For this reason, there is a growing concern for approaches that target to alleviate post-surgical pain. It is well-known that analgesic drugs (NSAIDs and corticosteroids) are widely used by the population; however, the use of such drugs should be minimized to avoid side effects and misuse (30). Likewise, the PBMT is an interesting approach since it could be applied immediately after the procedure and the benefits observed in our study showed to be satisfactory during the first 48 hours postoperative.

Compared to previous studies on the topic, the present randomized controlled trial has a bigger sample size (n=101 teeth), which provides higher statistical power to the analyses, increasing the possibility of external validation of the data. On the other hand, the use of a VAS to assess pain outcomes on the same patient in different third molar extractions could affect in some way the patient's responses and the study design did not prevent any self-medication by the patients.

## Conclusions

Photobiomodulation (PBMT) therapy presented a statistically significant reduction of postoperative pain scores when assessed 6, 24, and 48 hours after third molar extractions.
